# Long-term exposure to polycyclic musk tonalide – A potential threat to juvenile zebrafish (*Danio rerio*)?

**DOI:** 10.17221/40/2023-VETMED

**Published:** 2023-05-29

**Authors:** Jana Cahova, Jana Blahova, Lucie Plhalova, Petr Marsalek, Veronika Doubkova, Martin Hostovsky, Lenka Divisova, Jan Mares, Caterina Faggio, Zdenka Svobodova

**Affiliations:** ^1^Department of Animal Protection and Welfare and Veterinary Public Health, Faculty of Veterinary Hygiene and Ecology, University of Veterinary Sciences Brno, Brno, Czech Republic; ^2^Simulation Centre, Faculty of Medicine, Masaryk University, Brno, Czech Republic; ^3^Department of Zoology, Fisheries, Hydrobiology and Apiculture, Faculty of Agrosciences, Mendel University Brno, Brno, Czech Republic; ^4^Department of Chemical, Biological, Pharmaceutical and Environmental Sciences, University of Messina, Messina, Italy

**Keywords:** aquatic contamination, endocrine disruptor, oxidative stress, personal care products, vitellogenin

## Abstract

Polycyclic musk compounds are commonly used in personal care products to replace expensive natural fragrances. Due to their huge consumption, they have become a part of the aquatic environment. In the present study, a two-month exposure effect of tonalide on juvenile zebrafish (*Danio rerio*) was investigated. We determined the vitellogenin concentration to define the potential endocrine-disrupting effect of tonalide and also analysed selected indices to evaluate the induction of oxidative stress. The environmentally relevant concentration of tonalide (i.e., 500 ng/l) caused a significant decrease in the catalase activity (*P *< 0.05) and a significant increase (*P *< 0.05) in the lipid peroxidation. An increasing lipid peroxidation was also recorded for the highest concentration group tested (i.e., 50 000 ng/l). On the other hand, no significant changes were recorded in vitellogenin in all the exposed groups. Thus, based on these results, we have not demonstrated the endocrine-disrupting activity of tonalide in zebrafish. The results of the oxidative stress indices showed a significant impairment of the antioxidant defence after the two-month tonalide exposure, which could indicate part of the adaptive response to the tonalide toxicity.

In the last decades, personal care products, i.e., shampoos, sunscreens, detergents, perfumes etc., have been extensively used by millions of consumers. These products contain a wide range of chemicals that can get into the aquatic environment and affect non-target organisms (Kolarova et al. 2021). Musk compounds, as a part of common personal care products, are one of the most often found fragrances in the aquatic environment (Hodkovicova et al. 2020).

Polycyclic musk tonalide (AHTN) is a lipophilic compound with low water solubility and high *n*-octanol/water partition coefficient (K_ow _= 5.7). The proportion of musk chemicals in cosmetic products does not exceed 2% (Martinez-Giron et al. 2010), but due to the insufficient purification of musks in wastewater treatment plants and their lipophilic properties, these chemicals could persist in the water ecosystem and possibly bioaccumulate in the environment (Fang et al. 2017). AHTN has been found in the range of 82–49 904 ng/l in the influent wastewater from Tarragona, Spain (Vallecillos et al. 2015). AHTN can accumulate in the sludge particles from the wastewater plant due to its lipophilic properties and slow biodegradability. AHTN was detected in a range of 5.7–5 040 ng/g of dry weight (d.w.) in the sewage sludge from wastewater plants in Spain (Vallecillos et al. 2015) and in sediments at concentrations ranging from 1.4 ng/g to 36.6 ng/g of d.w. in the East China Sea (Hua et al. 2022). AHTN was found in the surface water in Italy in a range of 0.25–6 800 ng/l (Villa et al. 2012). AHTN was detected in many common aquatic species at concentrations reaching tens of ng/g of d.w. (Lyu et al. 2021).

Musk compounds have relatively low toxicity, but due to their ubiquity, it is necessary to examine their toxicity on non-target organisms. Therefore, the number of experiments studying the adverse effect of musk compounds on organisms has increased. One of the current topics in toxicology is the disruption of the antioxidant defence which could lead to an imbalance of the whole organism. Ehiguese et al. (2020) detected an increase in oxidative stress biomarkers in clams, *Ruditapes philippinarum*, after 21 days of AHTN exposure even at an environmental concentration. Hodkovicova et al. (2020) proved lipid peroxidation in juvenile rainbow trout after six weeks of AHTN exposure. Furthermore, AHTN induced antioxidant mechanisms and detoxifying enzymes in juvenile stages of zebrafish after 28 days of exposure (Blahova et al. 2018).

Endocrine disruption is another relevant topic of the toxicity of musks. Vitellogenin (VTG) is one of the most widely-used biomarkers for the detection of estrogenic chemicals. VTG is a precursor of egg yolk protein in females. The presence of the exogenous estrogenic compound induces VTG synthesis in male or juvenile fish. AHTN significantly induced the expression levels of hepatic VTG in male medaka, *Oryzias latipes* (Yamauchi et al. 2008), and in the sheepshead minnow (*Cyprinodon variegatus*) where the gene expression of *vtg1* in the yolk-sac was significantly downregulated (Ehiguese et al. 2021).

Based on the ubiquitous presence of AHTN in the aquatic environment and the possible negative effect on the non-target organisms mentioned in previous studies, we decided to evaluate the effect of AHTN in fish with an emphasis on the disruption of antioxidant defences representing an impairment of the organism’s balance, supplemented by an assessment of the xenoestrogenic potential of AHTN. The analysis of the oxidative stress indices consists of the determination of the antioxidant enzyme activities, such as catalase (CAT), glutathione peroxidase (GPx), glutathione reductase (GR), glutathione *S*-transferase (GST), and lipid peroxidation using a concentration of thiobarbituric acid reactive substances (TBARS). The estrogenic potential of AHTN was evaluated by analysis of the VTG in the whole-body homogenate of the male. These analyses were supplemented by a histopathological examination to observe any potential histological changes in the selected organs (i.e., muscles, gills, liver, kidneys, gonads, and intestine). To the best of our knowledge, only limited studies have investigated the effects of subchronic AHTN exposure in fish and we assume that the time of exposure is crucial for evaluating the AHTN toxicity. Therefore, we monitored the AHTN toxic effect after two-month of exposure in juvenile zebrafish (*Danio rerio*) to simulate natural conditions.

## MATERIAL AND METHODS

### Experimental design

The subchronic toxicity test was conducted according to the Organisation for Europe Economic Cooperation (OECD 2000) Guideline No. 215 Fish, Juvenile Growth Test (OECD 215). A total of 600 zebrafish (*Danio rerio*) at the age of 30 days were obtained from the experimental laboratory of Mendel University (Czech Republic). The fish were randomly distributed into twelve 20 l glass aquaria (i.e., 50 fish in each aquarium) and acclimatised for seven days before the toxicity test started. The whole experiment lasted for 8 weeks (56 days). The toxicity test was conducted in the approved facility of the Department of Animal Protection and Welfare and Veterinary Public Health (University of Veterinary Sciences Brno, Czech Republic).

The experimental fish were exposed to four different concentrations (50, 500, 5 000, and 50 000 ng/l) of AHTN (Sigma-Aldrich, St. Louis, USA; chemical purity 98%) in water. The lowest concentrations represent the environmental concentrations in the surface waters. Multiples of the tested concentrations were examined to demonstrate dose-dependent effects. A stock solution of AHTN was prepared by dissolving 10 mg of the chemical in 10 ml of acetone and then added to a 2-l volumetric flask with tap water (final concentration of 5 mg/l). This stock solution was prepared every day and an ultrasonic bath was also used to accelerate the dissolution. The stock solution was then dosed into the individual aquaria to achieve the required concentration. Due to the use of the solvent acetone for the stock solution, the concentration of this solvent in all the experimental groups was subsequently adjusted to be uniformly 0.005%. In addition, two control groups were included in the toxicity test. The first control group was with tap water only, while the second control group contained 0.005% of acetone as a solvent in addition to the tap water. The controls and all the tested concentrations were performed in duplicate. The experiment was conducted in a flow-through system, and the test solutions were replaced twice a day. The analysis of the AHTN concentration in the water samples was carried out using gas chromatography coupled with ion trap tandem mass spectrometry (GC/MS/MS). The methodology is described in detail by Blahova et al. (2018). The photoperiod interval of the experiment was 12 h light/12 h dark. The fish were fed with dried *Artemia salina* without shells at 8% body weight per day. The faecal material and uneaten food were removed from each aquarium daily by thorough bottom cleaning using suction. During the toxicity tests, the temperature, pH and oxygen saturation were measured daily using an HQ4300 Portable multi-meter (Hach, Loveland, USA), and the chemical parameters of the tap water were checked at 24-hour intervals in each aquarium, including recording the number of dead fish. The temperatures ranged from 23.9 °C to 24.7 °C; the oxygen content was not lower than 70%; and the pH ranged from 7.78 to 8.23. The basic chemical parameters of the water were as follows: acid neutralisation capacity (ANC_4.5_) – 4.2 mmol/l, chemical oxygen demand (COD) – 2.8 mg/l, nitrates (NO_3_^–^) – 23.5 mg/l, chlorides – 18.1 mg/l, Σ Ca^2+^ + Mg^2+^ – 3.1 mmol/l, total ammonia and nitrites (NO_2_^–^) were below the limit of determination (< 0.04 mg/l and < 0.02 mg/l, respectively).

At the end of the test, all the fish were euthanised using tricaine methanesulfonate (MS-222 at a concentration of 250 mg/l, for 10 min), and the total length and body weight of each fish were recorded. The euthanised fish were immediately processed for the histopathological examination and analyses of the selected oxidative stress indices and VTG concentration.

The experiment was approved by the Ethics Committee of the University of Veterinary Sciences Brno (Czech Republic) and by the Ministry of Education, Youth and Sports (Czech Republic). All the procedures complied with the national legislation – Act No. 246/1992 Coll., on the Protection of Animals Against Cruelty, as amended, and Decree No. 419/2012 Coll., on the Protection, Breeding and Use of Experimental Animals, as amended. Ethical approval No. MSMT-7412/2015-18 was authorised.

### Histopathological examination

The whole fish were fixed in buffered 10% neutral formalin. Subsequently, all the samples were dehydrated, embedded in paraffin wax, and sectioned on a microtome at a thickness of 4 μm. All the samples were stained with haematoxylin and eosin. The histology of the muscles, gills, liver, kidneys, gonads and intestine was determined using light microscopy (*n* = 6 in each experimental group).

### Analysis of the oxidative stress indices

The selected oxidative stress indices (CAT, GPx, GR, GST and TBARS) were determined in the whole-body samples which were stored in a deep-freeze box at –80 °C. Eight individual samples were analysed in each experimental group. At first, the whole-body samples were weighed, homogenised in a phosphate buffer containing 50 mmol/l potassium phosphate and 1 mmol/l ethylenediaminetetraacetic acid (EDTA) (pH 7.2, 1 : 10 w/v). The homogenate samples were divided into two portions. The first part of the homogenate was used for the analysis of the thiobarbituric acid reactive substances (TBARS) to evaluate the lipid peroxidation using malondialdehyde determination at 535 nm. The results of the lipid peroxidation are presented as nmol of TBARS per gram of tissue of wet weight (Lushchak et al. 2005). The second part of the homogenate was centrifuged (10 500 × *g*, 4 °C, 20 min) and the supernatant fraction was used for the analyses of the antioxidant and detoxifying enzyme activities.

The CAT activity was measured according to the methodology of Aebi (1984); the GPx activity was determined according to Flohe and Gunzler (1984); the GR activity was measured according to Carlberg and Mannervik (1975) and the GST activity was detected using Habig et al. (1974). The enzyme activities were related to the protein content (Smith et al. 1985). The determination of all the parameters was conducted spectrophotometrically using a Varioskan Flash Spectral Scanning Multimode Reader (Thermo Fisher Scientific Inc., Waltham, USA).

### Vitellogenin analysis

The quantitative analysis of the VTG content was analysed in the whole body samples using a commercial Enzyme-Linked ImmunoSorbent Assay kit for zebrafish *Danio rerio* (catalogue No.: V01008402-480; Biosense Laboratories, Bergen, Norway). Ten individual male samples were analysed in each experimental group. At first, the whole body sample was weighed, homogenised in a 50 mmol/l TRIS buffer (pH = 7.4, 1 : 4 w/v) with a 1% protease inhibitor and centrifuged (15 000 × *g* at 4 °C for 20 minutes). Supernatant fraction was stored at –80 °C before the final analysis. All the samples were kept on ice and were analysed in duplicate. Three different dilutions were used for the samples – 1 : 250; 1 : 2 500 and 1 : 25 000. An eleven-point calibration curve was prepared using the zebrafish VTG standard at concentrations from 0.12 ng/ml to 125 ng/ml. The calibration curve was performed for each microplate desk. The analysis followed the manufacturer’s instructions.

### Statistical analysis

The statistical analysis was performed using the statistical software Unistat v6.5 for Excel (London, UK). First, all the results were tested using the Shapiro-Wilk test and the Levene test for normality and homogeneity of variances across the groups, respectively. The experimental groups were always compared to the control group with acetone as a solvent, because no difference in all the indices was found between the control group with the tap water only and the control group with the solvent. The normally distributed data were subjected to a one-way analysis of variance (ANOVA), followed by the Tukey-HSD post-hoc test to determine the differences among the control and experimental groups. When the data did not meet the assumption of normality, a non-parametric multisample median test was applied. The difference was considered statistically significant when *P *< 0.05.

## RESULTS

### Mortality, behaviour and morphological indices

The mortality and behaviour of the fish were recorded every day. The mortality rate did not exceed 10% and the fish exhibited normal behaviour during the whole experiment. The morphological indices did not differ between the control and treated groups (data are not shown)

### Oxidative stress indices

The results of the antioxidant enzyme activities and lipid peroxidation are shown in Table 1. A significant decrease in the CAT activity was observed in the tested group exposed to 500 ng/l of AHTN. Significant increases in the TBARS concentrations were recorded in the groups treated with 500 ng/l and 50 000 ng/l of AHTN compared to the control. No statistically significant changes were detected in the GPx, GR and GST enzyme activities.

**Table 1:** Antioxidant enzyme activities and lipid peroxidation in the whole body homogenate of the zebrafish after subchronic exposure to tonalide

Indices	Control	Tested groups			
		50 ng/l	500 ng/l	5 000 ng/l	50 000 ng/l
CAT	151.9 ± 7.8^a^	125.2 ± 4.7^a^	**112.2 ± 6.0^b^**	125.9 ± 9.0^a^	128.6 ± 6.5^a^
GPx	31.0 ± 3.5^a^	26.1 ± 1.2^a^	24.7 ± 1.7^a^	32.1 ± 1.2^a^	25.8 ± 0.9^a^
GR	12.4 ± 0.8^a^	10.1 ± 0.7^a^	10.6 ± 0.5^a^	10.5 ± 0.6^a^	10.0 ±.0.3^a^
GST	176.2 ± 4.5^a^	177.9 ± 2.2^a^	188.1 ± 3.8^a^	186.0 ± 9.4^a^	157.7 ± 3.0^a^
TBARS	94.3 ± 15.7^b^	107.2 ± 15.6^b^	**261.9 ± 30.9^a^**	136.5 ± 14.5^b^	**261.4 ± 24.2^a^**

The data are presented as the mean ± standard error of the mean. Significant differences among the groups (*P *< 0.05) are indicated by different alphabetic superscripts and the bold font

CAT = catalase in μmol/min/mg protein; GPx = glutathione peroxidase in nmol/min/mg protein; GR = glutathione reductase in nmol/min/mg protein; GST = glutathione *S*-transferase in nmol/min/mg protein; TBARS = lipid peroxidation in nmol/g of tissue

### Vitellogenin analysis

The results of the VTG concentration are shown in Table 2. Vitellogenin was not detected in all the samples. In the cases where VTG was not detected, half the detection limit (i.e., 150 ng/ml) was used for the statistical analysis. No significant changes were recorded among the groups.

**Table 2:** Vitellogenin in the whole body homogenate of the male zebrafish after the tonalide exposure

Group	Number of analysed samples	% of positive samples	Range of detected concentrations (ng/ml)
Control	10	0	–
50 ng/l	10	0	–
500 ng/l	10	10	2 788^#^
5 000 ng/l	10	20	1 437–6 512
50 000 ng/l	10	30	497–8 966

^#^Only one positive sample was found

No significant changes were recorded among the groups (*P* > 0.05)

### Histopathological examination

The histopathological examination exposed only rare pathological changes (vacuolisation of hepatocytes and epithelial cells of renal tubules; chronic hepatitis). Considering these rare morphological changes, observed in both control groups and all the experimental groups, we assume they are not associated with the subchronic exposure to AHTN (data are not shown).

## DISCUSSION

AHTN, as a commonly used polycyclic musk, can be detected throughout the aquatic environment. Recent papers have mainly focused on the short-term exposure to AHTN, but it is very important to consider the subchronic effects on non-target aquatic organisms to simulate natural conditions. Our experiment revealed, after 56 days, various changes in the antioxidant protection of the zebrafish. The lowest concentration had no significant effect in the assessed indices, however, the higher concentration, which can be considered as environmentally relevant (i.e., detected in the sewage sludge and surface water in a more contaminated area) induced the antioxidant defence of the organisms and caused significant lipid peroxidation due to the massive free radical production. Considering the study by Blahova et al. (2018), where the catalase activity significantly increased at 5 000 and 50 000 ng/l of AHTN, we recorded the completely opposite trend, i.e., a decrease in the catalase activity after exposure to the 500 ng/l AHTN concentration. Catalase is one of the most important enzymes that secures the antioxidant resistance and stands in the first line of defence against oxidative stress. If we especially focused on fish, Chen et al. (2012) observed an increase in the catalase activity in goldfish (*Carassius auratus*) after 14 days’ galaxolide (musk) exposure. We assume that the inhibition of catalase in our experiment may be caused by the excessive production of reactive oxygen species. As our experiment lasted for two months, the significant reactive oxygen species production may have reduced the catalase activity for the 500 ng/l AHTN concentration. This fact would be supported by the results of TBARS concentration. The TBARS, as the by-product of lipid peroxidation, significantly increased in the same experimental group (i.e., 500 ng/l). The highest tested AHTN concentration did not cause any statistically significant changes for the catalase activity, we suppose that the presumable reason would be the re-producing/activating catalase. The TBARS significantly increased for the highest tested AHTN concentration, while the catalase activity did not change for this tested group. We assume that the whole compensation system is more complex and requires further investigation. However, the increasing content of TBARS could reflect the overproduction of oxidative radicals and could point to lipid peroxidation. This peroxidation could lead to damage to the cell lipid structures and even impair the DNA. Nevertheless, only rare changes on the organ structures were observed after the histopathological examination, thus impairment by the free radicals was not confirmed. Lipid peroxidation was also recorded in clams after 21 days’ AHTN exposure (Ehiguese et al. 2020). Considering fish, Blahova et al. (2018) revealed increased lipid peroxidation as well as the GST activity in juvenile zebrafish after 28 days exposure to AHTN at 50 000 ng/l and the GR activity was reduced at 50 ng/l. In contrast, the results of our experiment did not reveal any changes in the GST or GR activity. In view of all these facts, we suppose that the exposure time is decisive in assessing the antioxidant defence of the organism, as the decrease in CAT activity could demonstrate the consumption of the antioxidant capacity of the zebrafish. Although no significant histopathological changes were observed, prolonged exposure to this important micropollutant and the proven ongoing oxidative stress could lead to subsequent organ changes and damage to the whole organism.

Determination of the AHTN endocrine disrupting effect was one of the goals of our experiment. Ehiguese et al. (2021) posed that tonalide had an endocrine disrupting effect and revealed the downregulation of the *vtg1* gene in the yolk-sac larvae of the sheepshead minnow (*Cyprinodon variegatus*) after AHTN exposure. However, based on our results of the VTG analysis, we cannot confirm the endocrine disruption effect of AHTN in the juvenile zebrafish, even after two months of exposure. We assume that the life stage of the tested organism could be a decisive factor.

The results of our study contribute to the evaluation of the potential adverse effects of AHTN in fish. In our study, the subchronic exposure to AHTN caused a significant increase in the lipid peroxidation and a decrease in the catalase activity which could signify the impairment of the antioxidant defence even at the environmental relevant concentration (i.e., 500 ng/l of AHTN). Based on the VTG concentration result, we did not prove the endocrine disrupting effect of AHTN in the juvenile zebrafish. We assume that the time of exposure as well as the stage of the exposed individual are crucial for the assessment of the AHTN effects on the fish.

For the next experiment, we think that it would be interesting to evaluate the long-term exposure including the embryonic and larval stages to simulate the real conditions in the aquatic environment. It might be useful to include other markers, such as gene expression indices, in the assessment to confirm the adverse AHTN effect.
